# Trigeminal Neuralgia in an HIV Patient

**DOI:** 10.4103/0974-777X.59254

**Published:** 2010

**Authors:** Mohammad A Hashmi, Gautam Guha, Bibhuti Saha

**Affiliations:** *EKO CT & MRI Scan Centre, Medical College and Hospitals Campus, Kolkata, India*; 1 *Department of Neurology, Medical College and Hospitals, Kolkata, India*; 2 *School of Tropical Medicines, Kolkata, India*

**Keywords:** Human immunodeficiency virus, Trigeminal nerve, Magnetic resonance imaging (MRI)

## Abstract

Trigeminal neuralgia is a painful condition affecting face. Its commonest cause is the tortuous vessels in prepontine cistern. There are other causes also, like brainstem lesions and mass lesions, as well as inflammatory causes. We present a case of an HIV patient with marked involvement of trigeminal nerves, which is a unique finding in immunocompromised patients.

## INTRODUCTION

Trigeminal nerve is the largest of all the cranial nerves. It transmits sensory information from the face, oral and nasal cavities and most of the scalp and caries motor supply to the muscles of mastication. Disease involving nerve or adjacent to it can cause trigeminal neuralgia or loss of sensory or motor function in the distribution of the nerve. Disease affecting it can cause intense pain along its distribution. Neuropathy can affect the nerve from its origin in brainstem to its peripheral branches.[1] The nerve can be divided into four segments: brainstem, cisternal, Meckel's cave and cavernous sinus and extracranial course.[2] The commonest cause is vascular compression by tortuous vessel.[3] An inflammatory cause like meningitis can cause trigeminal neuralgia.

## CASE REPORT

**Figure 1:**
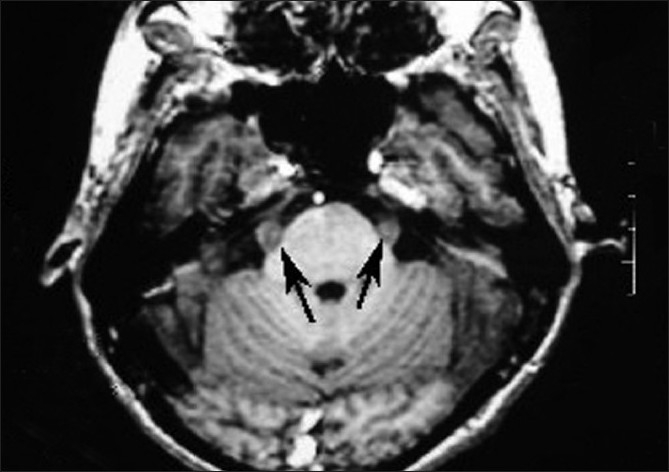
T1-weighted image showing thickened trigeminal nerves bilaterally as shown by arrows

**Figure 2:**
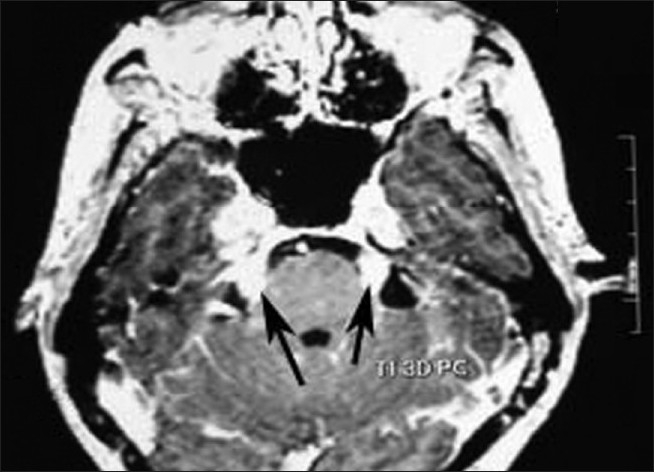
T1-weighted post-contrast images showing thickened and enhancing trigeminal nerves bilaterally as shown by arrows

An immunocompromised patient presented to us from the School of Tropical Medicine with pain around face, lips, eyes, scalp and forehead. The study was done on 1.5 tesla General Electronics signa. Contrast Gadodiamide (Omniscan) was used. Routine brain MRI was done. Plain MRI showed marked thickened trigeminal nerve bilaterally [Figure 1]. Post-contrast study showed marked enhancement of the above nerves [Figure 2].

## DISCUSSION

Trigeminal neuropathy can involve the whole nerve or part of the nerve from its origin to its peripheral branches.[1] Brainstem lesions affect mainly the nuclei of trigeminal nerves. Cerebrovascular causes are the commonest. The patient can have other symptoms. Demyelinating lesions like multiple sclerosis may affect brainstem.[2,4,5] Other white matter signals can also be seen in brain imaging. Mass lesions, mainly gliomas, involve brainstem.[2] Vascular malformation[6] and hamartomas are other conditions that affect brainstem. Viral rhombencephalitis can also affect brainstem.[7]

Tortuous vessel in prepontine cistern is considered to be the commonest cause of trigeminal neuralgia. Branches from superior cerebellar artery may cause pressure effect on the nerve.[3] Cerebellopontine-angle neoplasm's may cause neuropathy by compression on the nerve. Acoustic neuromas, meningiomas,[8] arachnoid cysts, epidermoid cysts[9] and metastatic lesions are found in this location.[2]

Meckel's cave or cavernous sinus lesions are meningioma, epidermoid tumor[10] and trigeminal neuroma.[1,2] Granulomatous or inflammatory diseases, such as neurosarcoid or tuberculosis, may involve the nerve or ganglion at this site. Vascular lesions like aneurysm can also cause pressure effect. Extracranial lesions can be mass lesion or any inflammatory condition.

Marked involvement of the trigeminal nerves in the above condition appears to be inflammatory and a combination of cisternal and Meckel's cave lesions. Sequelae of basal meningitis can affect cranial nerves.
